# The Effect of Filial Therapy on Depressive Symptoms of Children with Cancer and Their Mother’s Depression, Anxiety, and Stress: A Randomized Controlled Trial

**DOI:** 10.31557/APJCP.2019.20.10.2935

**Published:** 2019

**Authors:** Elaheh Ebrahimi, Hooshang Mirzaie, Mehrdad Saeidi Borujeni, Ghazal Zahed, Alireza Akbarzadeh Baghban, Navid Mirzakhani

**Affiliations:** 1 *Department of Occupational Therapy, School of Rehabilitation, *; 3 *Department of Child and Adolescent Psychiatry and Child Mental Health Center, Mofid Children Hospital, *; 4 *Protemics Research Center, Department of Basic Science, School of Rehabilitation, Shahid Beheshti University of Medical Sciences,*; 2 *Department of Occupational Therapy, University of Social Welfare and Rehabilitation Sciences, Tehran, Iran. *

**Keywords:** Childhood cancer, play therapy, filial therapy, parent, depression, anxiety, Stress

## Abstract

**Background:**

Childhood cancer is an overwhelming life event that can completely change the lives of the sufferers and their parents. Todays, advances of medical science have shifted the fetal nature of childhood cancer to chronic one exposing children and their family to behavioral and psychosocial problems. The aim of this study was to investigate the effect of filial therapy on children’s depressive symptoms and their mother’s stress, anxiety, and depression.

**Materials and Methods:**

In this randomized controlled trial, 32 mothers with their children who suffered from cancer were recruited (16 in each group). During a 10-week training sessions, filial therapy group underwent child-parent relation therapy (CPRT). Training sessions were held once a week. Control group received no training and only individual counseling sessions were held for them we needed. Both groups were assessed before and after the intervention using depression, anxiety, and stress questionnaire-21 (DASS-21), children depression inventory (CDI), and Wong-Baker faces pain rating scale (WBFPRS). Sample randomization and data analysis were conducted by using SPSS (version 20) and running independent t-test and chi-square test. P value< 0.05 was set as the significant level.

**Results:**

Mothers in the filial therapy group experienced significant decrease in their level of depression, anxiety, and stress in the post-test (p < 0.001). In contrast to filial therapy group, mothers in the control group did not show an improvement in their level of depression, anxiety, and stress. Moreover, the results of the current investigative showed that depression of children in the filial therapy group significantly reduced at post-test (p < 0.001). On the other hand, the mean of children’s depression in the control group remained steady.

**Conclusion:**

The findings of the present study revealed that using filial therapy could reduce the depression of children with cancer and their parent’s depression, anxiety, and stress. Accordingly, we suggest filial therapy programs as a routine for addressing psychosocial problems of children with cancer and their families.

## Introduction

Annually, 48 to 112 Iranian girls and 51 to 144 boys per million are diagnosed with cancer (Mousavi et al., 2010). Being diagnosed with this life-threating condition imposes a lot of stress on the sufferers and their families, especially their parents (Levi et al., 2000; Sloper 2000; Schweitzer et al., 2012; Heidarzadeh et al., 2014). Even though, with the advances of medical science, childhood cancer has been shifted from acute and fatal disease to chronic illness (Barakat et al., 1997; Vrijmoet-Wiersma et al., 2008), but it still can increase the risk of developing anxiety and depressive symptoms (Stuber, 1996), presenting both emotional (internalized symptoms) and behavioral problems (externalized symptoms) for the affected child and his/her (Sawyer et al., 2000; Tsai et al., 2013). Therefore, addressing psychological sequels for survivors and their parents attracted attentions of the researchers (Barakat et al.,1997). Parents of a chronically ill child spend significantly more of their time in care-taking activities, and they also report less intimacy, more parenting stress, less social support, more maternal depression, and increased strain on the role of parenting because of the additional duties that are imposed on them (Bolding and Llorens, 1991; Wood, 1991). Parents of child with cancer are exposed to anxiety and depression symptoms (Manne et al., 2001) and experience negative mood (Kazak et al., 2005). Depressive symptoms of the parent may interfere with the parent–child relationship and communication (Steele et al., 2004). Van der Geest et al., (2014) stated that changing dynamic between parent and child during initial treatment under the influence of parenting stress can affect their ability to parent their child in this stressful situation. Except anxiety and depression, parenting a child with cancer can also lead to higher levels of parenting stress (Kazak et al., 1997; Colletti et al., 2008). Some studies that investigated parenting stress have shown higher parenting stress in parents of a child with cancer compared with parents of physically disabled child (Hung et al., 2004). Based on these studies, this higher parenting stress and parents’ negative mood state are associated with behavior problems in children newly diagnosed with cancer (Fedele et al., 2011)

Besides to the problems that parents face, childhood cancer disturbs child’s daily roles and routines (Vrijmoet-Wiersma et al., 2008), limits him/her purposeful activities such as playing, and restricts his/her roles (Mohammadi et al., 2017). Play is one of the most important ways in which a child develops competence and mastery (Kramer 2018). Because of the lack of abstract thinking in children, which is a prerequisite for meaningful verbal expression and understanding of complex concepts and feelings, they more naturally express themselves through the concrete world of play and activity (Bratton et al., 2005). Todays, because of children’s varied developmental needs, play therapy is widely used to treat emotional and behavioral problems (play as a means) or to develop skills and abilities required for performing daily activities (play as an end) (Bratton and Landreth, 1995; Parham and Fazio, 2008).

While there are many different play therapy approaches available, filial therapy because it is taught to the parents and it is done by the parents. In addition, filial therapy is structured to enhance and strengthen the relationship between the parent and the child (Bratton and Landreth, 1995; Jang, 2000). Therefore, it may have a better effect on child with cancer and his/her parent. A meta-analysis compared the effect of filial therapy and play therapy on children and concluded that filial therapy could exert better treatment effect than play therapy (Bratton et al., 2005).

Filial therapy is based on training basic play therapy techniques to parents, and it was developed by Bernard and Louise Guerney in the early 1960s. In this specific play therapy approach, parents or one parent (most often the mother) are taught the elements of play therapy to develop skills in reflecting feelings, in accepting their child’s feelings, and in setting limits on the child’s behavior in a caring and non-punitive fashion (Guerney, 1964; Bratton and Landreth, 1995). Based on the work of Guerneys and Landreth (1991), a more condensed parent training format was developed, which is known as child parent relationship therapy (CPRT) (Landreth, 2012). CPRT is based on the rationale that the relationship between parent and child is an essential and curative therapeutic dimension for improving and removing children’s problems and preventing the development of these childern’s future problems (Landreth et al., 2006). Landreth’s 10-session filial therapy training protocol emphasizes a balance of didactic and supervision experiences in a 2-hr weekly support group format and requires parents to videotape their weekly play sessions at home (Landreth and Lobaugh, 1998; Landreth et al., 2006; Landreth, 2012). In each session, therapist trains parents (usually mothers) and discuss about one of the CPRT features such as parent-child play session skills and procedures, then mothers do what they learned during a 30-minute session with their child. In next session, mothers and therapist discuss about previous content and the feedback they received from playing with their child, then they train new feature. In the special playtimes, the child is allowed to lead the play. The parent does not initiate topics of conversation, content of play, how the time will be spent, or offer suggestions for solutions to problems. The child leads the 30minute special playtime and the parent follows him/her (Landreth et al., 2006).

In this study, we aimed to investigate the effect of filial therapy on cancer children’s depressive symptoms and their mother’s stress, anxiety and depression.

## Materials and Methods

This single-blind randomized control trial was performed using pre- and post-test design to investigate the effects of 10-session filial therapy on cancer children’s depressive symptoms and their mother’s stress, anxiety and depression. The statistical population included two groups of experimental (EG) (n=16) and control (CG) (n= 16).

**Figure 1:**
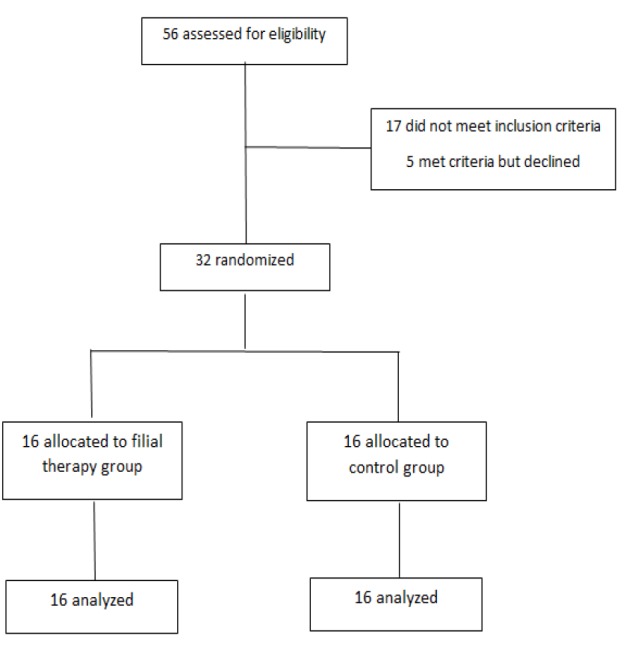
CONSORT Flowchart of the Study Process

**Table 1:** Demographics and Participants’ Characteristics

Variable	Filial Therapy	Control	P
Children’s age	9.25 years (±0.56)	9.12 years (±1.74)	0.83
Mother’s age	36.50 years (±5.15)	38.62 years (±7.23)	0.34
Child's age at the time of diagnosis	7.78 years (±1.32)	7.37 years (±1.89)	0.48
Wong-Baker	8.31 (±1.25)	7.75 (±1.18)	0.2
Duration of disease	1.47 years (±1.61)	1.75 years (±0.85)	0.25
Mother's education	Diploma= 12	Diploma= 14	0.5
	Bachelor= 3	Bachelor= 2	
	Master= 1	Master= 0	
Mother active play with child	Yes= 7	Yes= 8	0.72
	No= 9	No= 8	
Child active play whit mother	Yes= 8	Yes= 7	0.72
	No= 8	No= 9	

**Table 2:** Mean of CDI and DASS at Testing Sessions

Variable	Group	Pre-test	Post-test	p	t	95% CI	
						Lower	Upper
CDI	Filial Therapy	25.19 (±3.52)	4.44 (±1.86)	0	26.88	19.1	22.39
	Control	25.25 (±4.64)	27.63 (±5.35)	0.11	−1.67	8.77	13.6
DASS (depression)	Filial Therapy	12.63 (±3.84)	1.44 (±1.71)	0	9.9	7.38	13.36
	Control	14.44 (±4.09)	15.49 (±3.27)	0.12	−1.63	9.81	14.68
DASS (anxiety)	Filial Therapy	13.31 (±5.42)	2.94 (±1.91)	0	7.38	−5.40	0.65
	Control	14.69 (±5.01)	15.12 (±4.19)	0.78	−0.28	−2.88	0.38
DASS (stress)	Filial Therapy	16.19 (±3.65)	3.94 (±1.79)	0	10.72	−3.78	2.9
	Control	16.94 (±5.06)	17.39 (±2.98)	0.69	−0.41	−2.73	1.85

**Table 3:** Between Group Differences in Mean Change Scores for CDI and DASS

	Mean change: Pre-test to Post-test						
	Filial Therapy	Control	t	df	p	95% CI	
						Lower	Upper
CDI	−20.75 (±3.08)	2.37 (±5.69)	−14.287	30	0.001	−26.43	−19.81
DASS (depression)	−11.18 (±4.51)	1.25 (±3.60)	−9.109	30	0.001	−15.22	−9.64
DASS (anxiety)	−10.37 (±5.61)	0.43 (±6.28)	−5.131	30	0.001	−15.11	−6.50
DASS (stress)	−12.25 (±4.56)	0.43 (±4.30)	−8.086	30	0.001	−15.89	−9.48

*Design and Sampling*

Approval was obtained to conduct this research from Ethics Committee and Pediatric Congenital Hematologic Disorders Research Center of Shahid Beheshti University of Medical Sciences (IR.SBMU.REC.1396.646). Announcement was made through papers distributed in Mofid children’s hospital. Participants were mothers of children suffering from cancer and were treated as outpatients at the hospital. Eligibility criteria were established based on previous studies in this regard: be parent of a chronically ill child and age between 6 to 12 years, . Inclusion criteria were as follows: be willing to participate in the study, experiencing symptoms of depression, anxiety, and stress based on depression anxiety stress scales questionnaire (DASS), observing symptoms of depression in her child based on Children’s Depression Inventory (CDI), obtaining mini-mental state evaluation score ≥24, and be able to speak and read in Persian. Exclusion criteria were as follows: exacerbation of the child’s disease or die, irregular presence of the mother at the training sessions, unwillingness to continue the study.

The sample size for the trial was determined based on previous studies (Zareapour et al., 2009; Alavian et al., 2016) using a significance level of .05, β= 0.1, and a power of 90%. Anticipating a drop-out rate due to the nature of childhood cancer and considering Landerth standard groups of filial therapy consisting of eight people, however, we planned to enroll 32 participants (16 in each group).

Randomization was performed twice for parents of boys and girls separately with SPSS software, so that numbers 1 to 16 according to different boys and girls were enrolled to the software; and by random sampling subtest, eight boys (and eight girls in next time) elected, then parent (generally mother) of that child assigned to the experimental group and others to the control group.

All patients provided written informed consent. They were assured that they were going to participat in a noninvasive experiment with the aim of improving their relationship with their child and reducing the level of stress and depression in them and their children. They were also assured about the confidentiality of their information.

*Procedures*

This study was conducted from December 2017 to July 2018 on mothers of childern with cancer who received outpatient chemotherapy at Mofid children’s hospital affiliated with Shahid Beheshti University of Medical Science, in Tehran, Iran. In order to ensure the eligibility of participants, both mothers and their children completed DASS and CDI questionnaires. Then, eligible mothers were randomly allocated into filial therapy group and control group. Filial therapy group was subdivided into two equal groups in order to hold group filial therapy sessions. Next, family-centered play therapy guide booklet included techniques and homework were provided to filial therapy group. In this booklet, 16 general rules are introduced about child-parent relation consisting of tips to guide parents to learn how to communicate with child, deal with the child correctly, limit, supervise, transfer responsibility, and teach him/her decision making. In addition, limits about generalization of those trainings out of play situation were explained to mothers. Then, these rules and techniques were taught to mothers by a therapist (see Table 1 for more information about content of each session). Mothers were asked to play with their child one time per week in a restricted place with the lowest distraction stimulus, without fear of breakable objects, and at a quiet place, and try to observe what they had learned during the training sessions. Mothers had to record their plays and also write down the time and place of their plays in the guide booklet. At the beginning of the next session, the content of the previous session was reviewed; mothers discussed about their experiences, and gave feedbacks they had received from the play with their child.

For the control group, no intervention was provided. Only individual counseling sessions were held for this group. These sessions concerned about incidence of anxiety in parents after confronting childhood cancer diagnosis, parental stress and negative mood and impact of these stresses and negative mood on parenting and child caring duties that may cause internalized behavioral problems and somatic complaints for children. In these sessions, the filial therapy and its techniques were not taught so they were not aware of the experimental group’s training. Parents in the control group were offered filial therapy training after the study.

*Instruments*

Before conducting the intervention, both groups were undergoing pre-test assessment. The post-test was performed the day after the end of the intervention (after 5 weeks). All assessments were performed by the same examiner, who was blinded about the objective of the study.

In this study, we used Persian version of the DASS-21, a short form of the DASS-42 to investigate the depression, anxiety and stress of mothers before and after the intervention. It is a self-report measure consists of 21 items comprising 3 subscales of 7 items. Each item measures the extent to which each state has been experienced over the past week, rated on a 4 point Likert scale from 0 which means “did not apply to me at all” to 3 “applied to me very much or most of the time”. The depression subscale utilizes items which largely assess dysphoria, anhedonia, hopelessness, devaluation of life, and inertia; the Anxiety subscale assesses acute responses of fear as well as somatic and subjective symptoms of anxiety; and the stress subscale contains items which measure tension, agitation, irritability, and difficulty in relaxing (Henry and Crawford, 2005). Asghari et al., (2008) examined the factor structure, convergent and discriminant validity, and reliability of the Persian DASS-21 amongst a non-clinical Iranian population and reported that the Persian version of the DASS-21 had satisfactory psychometric properties and can be used amongst the Iranian adult population.

To assess depressive symptoms of children with cancer, CDI was used. CDI is a 27-item, a self-report measure. To complete CDI, the child is asked to endorse the one of three descriptions that best applies to him or her during the last 2 weeks (e.g., “I feel like crying every day,” “I feel like crying many days,” “I feel like crying once in a while”). Responses are scored on a 0-2 scale, with 2 representing the severe form of a depressive symptom and 0 representing the absence of that symptom. Dehshiri et al., (2009) investigated primary psychometric properties of the Persian version of CDI and assured the reliability of total CDI and its internal consistency (0.82 and 0.83, respectively).

We also used Wong-Baker Faces Pain Rating Scale (WBFPRS) to evaluate children’s pain. The WBFPRS is a horizontal scale of 6 hand-drawn faces, now scored from 0 to 10, that range from a smiling (“no hurt”) face on the left to a crying (“hurts worst”) face on the right (Wong and Baker, 1988; Tomlinson et al., 2010).

*Statistical analysis*

In order to assess any potential differences between groups, demographic characteristics were compared between groups using independent t-test and chi-square test. To test the study hypothesis, first we computed differences of Baseline and post-test for mother’s depression, anxiety, and stress scores as well as CDI scores. Then, independent t test was used to compare and analyze changes between two groups during study period. In addition, paired t test was used to investigate changes within groups at pre- and post-test. All analyses were computed using SPSS (version 20) and considering α level set at p < 0.05. Statistical analysis was performed by a biometrics blinded to the study.

## Results

Thirty two patients were randomized to the filial therapy group (n = 16) and the control group (n=16); Mean age of the participants and their children’s in the filial therapy group was 36.50 ±5.15 years old and 9.25 ±0.56 years old, respectively and it was 38.62 ±7.23 years old and 9.12 ±1.74 years old in the control group, respectively. At baseline, there were no significant differences between two groups in terms of demographic and clinical characteristics or outcome measures (Tables 1 and 2).

Analysis of CDI scores using paired t-test revealed significant decreases in CDI scores during the study in the filial therapy group (t= 26.88, p= 0.000), but not in the control group (t= −1.67, p= 0.11) (Table 3).To compare the mean changes between groups, independent t-test was run, and the results revealed a significantly higher changes in CDI scores for filial therapy group compared to the control group that remained almost steady at the post-test session (filial therapy group: = −20.75 and control group: = 2.37. p= 0.001).

In addition, analysis of DASS scores, which measures depression, anxiety and stress and presents separate score for each subscales, showed significant changes in depression, anxiety, and stress of mother’s in the filial therapy group (t= 9.90, p= 0.000, t= 7.38, p= 0.000, and t= 10.72, p= 0.000), respectively. On the other hand, no significant difference was observed in this regard in the control group (Table 2). Independent t-test results indicated significantly higher reduction in filial therapy group compared to the control group at the post-test concerning DASS depression scores (filial therapy group: = −11.18 vs. control: = 1.25); DASS anxiety scores (filial therapy group: = −10.37, control: = 0.43), and DASS stress scores (filial therapy group: = −12.25, control: = 0.43) (Table 3).

## Discussion

The aim of this study was to investigate the effect of filial therapy on children’s depressive symptoms and their mother’s stress. To achieve this aim, we used DASS questionnaire. Result of this study showed that filial therapy can be a feasible and efficient method to deal with children suffering from cancer and their mothers since it significantly reduced children’s depression and mother’s depression, anxiety, and stress. Mothers in the filial therapy group gained significantly lower scores than mothers in the control group regarding depression, anxiety, and stress. However, the level of depression, anxiety, and stress of mothers in the control group remained essentially unchanged over the period of 10 weeks. The results of the DASS suggested that filial therapy training was effective in decreasing overall parent’s depression, anxiety, and stress. Furthermore, comparison of children in two groups, based on the CDI scores, revealed significantly differences between to group at post-test while the two groups were initially equal in terms of severity of depression. However, the mean of depression level of children in the control group remained steady during the study. Although main features of depression in children are same as adults, their expressions vary according to the age of the children (American Psychiatric Association, 2013). Some of the most common symptoms of depression in children such as feeling of hopelessness, irritability or anger, feelings of worthlessness or guilt, and uncontrollable outbursts of crying can be greatly affected by the play (Li et al., 2016). This can be a reason for reduction of children’s depression scores in the present study.

We found no published articles on the efficacy of filial therapy on children with cancer; therefore, we could not compare our findings with those of other studies. To the best of our knowledge, we were the first to investigate the effects of filial therapy on depressive symptoms of children with cancer. Alavian et al., (2016) used CPRT method to design self-made plays for reducing depression symptoms in children with cancer and their parent’s perceived stress, but the authors did not exactly follow the principles and rules of the filial therapy. They had held eight play sessions in only two weeks in a hospital. Their results showed that CPRT could reduce depression symptoms of children, but it did not have any effects on the stress of mothers (Alavian et al., 2016). This discrepancy may be because of short period of treatment in the aforementioned study. Mohammadi et al., (2017) investigated the effect of play-based occupational therapy on pain, anxiety, and fatigue level of inpatient children with cancer. They reported that play therapy could reduce the anxiety of hospitalized children with cancer (Mohammadi et al., 2017). Though the effect of filial therapy on children with cancer has not been investigated until now; if we consider child-hood cancer as a chronic illness, two studies are available for the comparison. Glazer-Waldman et al., (1992) for the first time studies the efficacy of filial therapy as an intervention for families with chronically ill children. They indicated an increase in parental acceptance of their children disease. Although child anxiety did not change significantly, they claimed that the small number of participants could be attributed to this finding. In another study, Tew et al., (2002), conducted a 10-week filial therapy training and reported that parents in the experimental group experienced significantly reduced level of stress as compared to parents in the control group. This study also addressed the issue of parental acceptance of their child’s disease and the conditions induced by the disease , and suggested filial therapy training as an effective method to help parents become more inclined to accept their children disease.

Results of our study are in line with those of other studies on the effectiveness of filial therapy in reducing parental stress. In addition, we found that filial therapy could affect depression and anxiety of parents, too. Nevertheless, follow-up studies are needed to determine the long-term effects of filial therapy on depression, anxiety, and stress of child with cancer as well as their parents. Finally, although there are few studies on the effect of filial therapy on cancer or chronically ill children, its efficacy was tested in children populations in general and one meta-analysis stated that filial therapy was able to produce larger treatment effect than play therapy.

In conclusion, the result of this study suggested the 10-week filial therapy training model as an effective intervention for child with cancer and their parents. During this 10-week course, mothers learned the basic skills. Videotapes showed that mothers were a bit confused in the first two or three sessions; but over time, they could demonstrate these learned skills in 30-minute play session with their child. Mothers reported that they were able to cope more appropriately with their child’s condition and noticed a positive change in their children. The results of this study also showed the therapeutic value of play sessions based on child-centered play therapy procedures, and suggested teaching play skills to parents with cancer to reduce depressive symptoms of their children and to relive their own depression, anxiety and stress. 

*Limitations*

One of the limitations of this study was that we could not provide a standard treatment for the control group, which could be one of the reasons for the notable differences between two groups at post-test regarding DASS and CDI scores. Another limitation of the current investigation was that some mothers did not take a video of a few sessions or at all for cultural or technical reasons although mothers were asked to take a video of playing with their child. We had two cases of drop-out in filial therapy group, one because of the mother’s reluctance to continue and another because of exacerbation of child condition which was replaced with new cases. As a result, we had to hold their sessions apart from others. Finally, lack of doing follow-up limited the generalization of our results. Further studies with follow-up are recommended determining the long-term effects of filial therapy on stress, anxiety, and depressive symptoms of children with cancer and their parents.

## References

[B1] Alavian RS, Tabibi Z, BaniHashemi A, AbdeKhodaee MS (2016). The effectiveness of parent-child play therapy on decreasing depression symptoms in children with cancer, decreasing perceived stress on their mothers and improving parent-child relationship [in Persian]. J Fam Res.

[B2] Asghari A, Saed F, Dibajnia P (2008). Psychometric properties of the Depression Anxiety Stress Scales-21 (DASS-21) in a non-clinical Iranian sample. Int J Psychol.

[B3] American Psychiatric Association (2013). Diagnostic and statistical manual of mental disorders (DSM-5®).

[B4] Barakat LP, Kazak AE, Meadows AT (1997). Families surviving childhood cancer: A comparison of posttraumatic stress symptoms with families of healthy children. J Pediatr Psychol.

[B5] Bolding DJ, Llorens LA (1991). The effects of habilitative hospital admission on self-care, self-esteem, and frequency of physical care. Am J Occup Ther.

[B6] Bratton S, Ray D, Rhine T, Jones L (2005). The efficacy of play therapy with children: A meta-analytic review of treatment outcomes. Prof Psychol Res Pract.

[B7] Bratton S, Landreth G (1995). Filial therapy with single parents: effects on parental acceptance, empathy, and stress. Int J Play Ther.

[B8] Colletti CJ, Wolfe Christensen C, Carpentier MY (2008). The relationship of parental overprotection, perceived vulnerability, and parenting stress to behavioral, emotional, and social adjustment in children with cancer. Pediatr Blood Cancer.

[B9] Dehshir GH, Najafi M, Shikhi M, Habibi Askarabd M (2009). Investigating primary psychometric properties of children’s depression inventory (CDI). J Fam Res.

[B10] Fedele DA, Mullins LL, Wolfe-Christensen C, Carpentier MY (2011). Longitudinal assessment of maternal parenting capacity variables and child adjustment outcomes in pediatric cancer. J Pediatr Hematol Oncol.

[B11] Glazer-Waldman HR, Zimmerman JE, Landreth G, Norton D (1992). Filial therapy: An intervention for parents of children with chronic illness. Int J Play Ther.

[B12] Guerney B (1964). Filial therapy: Description and rationale. Consult Psychol J.

[B13] Heidarzadeh M, Rassouli M, Mohammadi Shahbolaghi F (2014). Posttraumatic growth and its dimensions in patients with cancer. Middle East J Cancer.

[B14] Henry JD, Crawford JR (2005). The short-form version of the Depression Anxiety Stress Scales (DASS-21): Construct validity and normative data in a large non-clinical sample. Br J Clin Psychol.

[B15] Hung JW, Wu YH, Yeh CH (2004). Comparing stress levels of parents of children with cancer and parents of children with physical disabilities. Psychooncology.

[B16] Jang M (2000). Effectiveness of filial therapy for Korean parents. Int J Play Ther.

[B17] Kazak AE, Boeving CA, Alderfer MA, Wei-Ting H, Reilly A (2005). Posttraumatic stress symptoms during treatment in parents of children with cancer. J Clin Oncol.

[B18] Kazak AE, Barakat LP (1997). Brief report: Parenting stress and quality of life during treatment for childhood leukemia predicts child and parent adjustment after treatment ends. J Pediatr Psychol.

[B19] Kramer P (2018). Frames of reference for pediatric occupational therapy.

[B20] Landreth G (2012). Play therapy: The art of the relationship.

[B21] Landreth G, Bratton S, Kellam T, Blackard S (2006). Child Parent Relationship Therapy (CPRT) treatment manual: A 10-session filial therapy model for training parents.

[B22] Landreth G, Lobaugh A (1998). Filial therapy with incarcerated fathers: Effects on parental acceptance of child, parental stress, and child adjustment. J Couns Dev.

[B23] Levi R, Marsick R, Drotar D, Kodish E (2000). Diagnosis, disclosure, and informed consent: learning from parents of children with cancer. J Pediatr Hematol Oncol.

[B24] Li WH, Chung JK, Ho KY, Kwok MC (2016). Play interventions to reduce anxiety and negative emotions in hospitalized children. BMC Pediatr.

[B25] Manne S, Nereo N, DuHamel K (2001). Anxiety and depression in mothers of children undergoing bone marrow transplant: Symptom prevalence and use of the Beck Depression and Beck Anxiety Inventories as screening instruments. J Consult Clin Psychol.

[B26] Mohammadi A, Mehraban AH, Damavandi S (2017). Effect of play-based occupational therapy on symptoms of hospitalized children with cancer: A single-subject study. Asia Pac J Oncol Nurs.

[B27] Mohammadi A, Mehraban AH, Damavandi S, Alizadeh Zarei M, Amini M (2017). Participation in daily life activities among children with cancer. Middle East J Cancer.

[B28] Mousavi SM, Pourfeizi A, Dastgiri S (2010). Childhood cancer in Iran. J Pediatr Hematol Oncol.

[B29] Parham LD, Fazio LS (2008). Play in occupational therapy for children. Mosby.

[B30] Sawyer M, Antoniou G, Toogood I, Rice M, Baghurst P (2000). Childhood cancer: A 4-year prospective study of the psychological adjustment of children and parents. J Pediatr Hematol Oncol.

[B31] Schweitzer R, Griffiths M, Yates P (2012). Parental experience of childhood cancer using Interpretative Phenomenological Analysis. Psychol Health.

[B32] Sloper P (2000). Predictors of distress in parents of children with cancer: A prospective study. J Pediatr Psychol.

[B33] Steele RG, Dreyer ML, Phipps S (2004). Patterns of maternal distress among children with cancer and their association with child emotional and somatic distress. J Pediatr Psychol.

[B34] Stuber.ML (1996). Psychiatric sequelae in seriously ill children and their families. Psychiatr Clin North Am.

[B35] Tew K, Landreth G, Joiner KD, Solt MD (2002). Filial therapy with parents of chronically ill children. Int J Play Ther.

[B36] Tomlinson D, Von Baeyer CL, Stinson JN, Sung L (2010). A systematic review of faces scales for the self-report of pain intensity in children. Pediatrics.

[B37] Tsai MH, Hsu JF, Chou WJ (2013). Psychosocial and emotional adjustment for children with pediatric cancer and their primary caregivers and the impact on their health-related quality of life during the first 6 months. Qual Life Res.

[B38] Van der Geest IM, Van den Heuvel Eibrink MM, Passchier J (2014). Parenting stress as a mediator of parents’ negative mood state and behavior problems in children with newly diagnosed cancer. Psychooncology.

[B39] Vrijmoet-Wiersma J, Van Klink J, Kolk AM (2008). Assessment of parental psychological stress in pediatric cancer: A review. J Pediatr Psychol.

[B40] Wong DL, Baker CM (1988). Pain in children: comparison of assessment scales. Pediatr Nurs.

[B41] Wood TA (1991). A comparison of stress and marital adjustment with families of chronically ill, handicapped and non-ill/non-handicapped children. J Spec Educ.

[B42] Zareapour A, Falahi Khoshknab M, Kashaninia Z, Biglarian A, Babashahabi R (2009). Effect of group play therapy on depression in children with cancer. Sci J Kurdistan Univ Med Sci.

